# Photodynamic therapy‐induced precise attenuation of light‐targeted semicircular canals for treating intractable vertigo

**DOI:** 10.1002/SMMD.20230044

**Published:** 2024-10-19

**Authors:** Yingkun Yang, Tong Zhao, Feixue Mi, Hongzhe Li, Pingbo Huang, Fangyi Chen

**Affiliations:** ^1^ Department of Biomedical Engineering Southern University of Science and Technology Shenzhen Guangdong China; ^2^ Division of Life Science Hong Kong University of Science and Technology Hong Kong China; ^3^ Department of Otolaryngology‐Head and Neck Surgery Stanford University Stanford California USA; ^4^ Research Service VA Loma Linda Healthcare System Loma Linda California USA; ^5^ Department of Otolaryngology‐Head and Neck Surgery Loma Linda University Health Loma Linda California USA; ^6^ State Key Laboratory of Molecular Neuroscience Hong Kong University of Science and Technology Hong Kong China; ^7^ Hong Kong Branch of Guangdong Southern Marine Science and Engineering Laboratory (Guangzhou) Hong Kong University of Science and Technology Hong Kong China; ^8^ Guangdong Provincial Key Laboratory of Advanced Biomaterials Southern University of Science and Technology Shenzhen China

**Keywords:** horizontal semicircular canal, intractable vertigo, photodynamic therapy, targeted therapy, vestibular apparatus

## Abstract

Vertigo is a common symptom of various diseases that affects a large number of people worldwide. Current leading treatments for intractable peripheral vertigo are to intratympanically inject ototoxic drugs such as gentamicin to attenuate the semicircular canal function but inevitably cause hearing injury. Photodynamic therapy (PDT) is a noninvasive therapeutic approach by precisely targeting the diseased tissue. Here, we developed a PDT‐based method for treating intractable peripheral vertigo in a mouse model using a polymer‐coated photosensitizer chlorin e6 excited by red light. We found that a high dose of PDT attenuated the function of both semicircular canals and otolith organs and damaged their hair cells. Conversely, the PDT exerted no effect on hearing function or cochlear hair‐cell viability. These results suggest the therapeutic potential of PDT for treating intractable peripheral vertigo without hurting hearing. Besides, the attenuation level and affected area can be precisely controlled by adjusting the light exposure time. Furthermore, we demonstrated the potential of this therapeutic approach to be minimally invasive with light irradiation through bone results. Thus, our PDT‐based approach for attenuating the function of the semicircular canals offers a basis for developing a less‐invasive and targeted therapeutic option for treating vertigo.

## Highlights

- Developed a novel method based on photodynamic therapy (PDT) to precisely injure the vestibular hair cells for treating severe vertigo without hurting cochlear hair cells.
- Applied both vestibulo‐ocular reflex (VOR) and off‐vertical‐axis‐rotation (OVAR) tests to precisely localize and quantify the injury in mice.
- Investigated the minimal‐invasive surgical approach for applying PDT.

## Graphical abstract

Treatment of peripheral vestibular vertigo, such as intratympanic gentamicin, usually leads to serious complications such as hearing loss due to the lack of specificity on the vestibular hair cell injury. In this article, the authors developed a PDT‐based method to precisely injure the vestibular hair cells. They found that a high dose of PDT attenuated the function of both semicircular canals and otolith organs without cochlear hair cell damage.

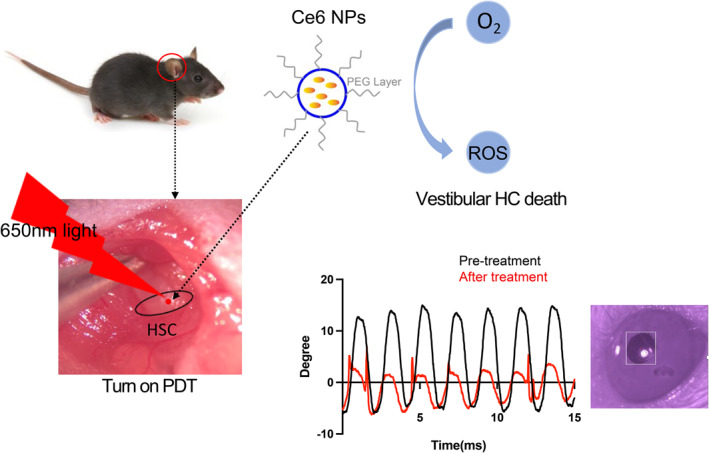

## 1 INTRODUCTION

Vertigo affects a large number of people worldwide, with a lifetime prevalence of 7.4%, a 1‐year prevalence of 4.9%, and an incidence of 1.4%.^1^ Vertigo is not a single and specific disease but rather a common symptom of various diseases. The main symptom of vertigo is a sensation of spinning or tilting while stationary, and vertigo is also frequently associated with nausea, vomiting, and loss of balance.^2^, ^3^ Vertigo can be classified into peripheral and central vertigo: While peripheral vertigo results from dysfunction of the part of the inner ear that controls balance, such as the semicircular canals or vestibular nerve, central vertigo is caused by injury or dysfunction in the balance centers of the central nervous system.^4^ Notably, peripheral vertigo accounts for ∼80% of all cases of vertigo.

Peripheral vertigo (also known as ontological or vestibular vertigo) is caused by diverse conditions that affect the function of the semicircular canals or vestibular nerve, including benign paroxysmal positional vertigo (BPPV, 32%), Meniere disease (MD, 12%), labyrinthitis, and other diseases. However, the treatment of peripheral vertigo presents various challenges due to the nonspecific etiologies involved and the complex anatomic structure of the inner ear. For treating the mild symptoms of peripheral vertigo, physicians first consider nonsurgical procedures or medicine administration, such as the canalith repositioning procedure to reposit loosened otoliths^5^ and the use of drugs such as diazepam to suppress vestibular function.^6^ Conversely, persistent severe vertigo is typically treated using clinical surgeries such as endolymphatic sac surgery,^7^ semicircular canal occlusion,^8^ and vestibular neurectomy or destructive procedures such as intratympanic gentamicin injection (ITGI).^9^, ^10^ Most of these therapies are based on the inhibition of vestibular hair cells to suppress vestibular function. However, these surgical or destructive treatments are highly invasive and can lead to serious complications such as hearing loss.^11^

Photodynamic therapy (PDT) is a noninvasive therapeutic approach that has been used for treating several diseases, such as skin tumors and head and neck tumors,^12^, ^13^ due to its high temporal and spatial specificity and minimal invasion of nontargeted tissues. PDT features three essential components: a nontoxic photosensitizer as the drug, light of an appropriate wavelength to excite the photosensitizer, and oxygen surrounding the target tissues.^14^, ^15^ Excitation of the photosensitizer by the specific light treatment converts the surrounding oxygen into reactive oxygen species (ROS) to damage biological tissues.

To avoid the invasiveness and potential damage of hearing function associated with current surgical and destructive treatments for intractable peripheral vertigo, we used the principle of PDT to achieve precise and tunable injury of the semicircular canals and otolith organs. Furthermore, the injury could be limited to within the semicircular canals by tuning down the PDT level. Attenuating the function of vestibular organs represents an effective method to relieve vertigo symptoms. Our innovative approach can potentially be used for treating intractable peripheral vertigo and provides a superior alternative to current invasive treatments of intractable vertigo, such as ITGI and semicircular canal occlusion.

## 2 MATERIALS AND METHODS

### 2.1 Chemicals and antibodies

Poly(ethylene glycol) methyl ether block‐poly(lactide‐co‐glycolide) (PEG‐PLGA; PEG, M_n_ 2000; PLGA, M_n_ 4500; Cat. # 764825), rose bengal (RB; Cat. # 330000), 9,10‐anthracenediyl‐bis (methylene) dimalonic acid (ADMA; Cat.# 307554‐62‐7), ampicillin (Cas 69‐52‐3), and FM1‐43 (Cat. # T3163) were purchased from Sigma; the photosensitizer chlorin e6 (Ce6; Cat. # 19660‐77‐6) from Frontier Scientific; rabbit anti‐myosin 7a from Proteus Biosciences (Cat. # 25–6790); Alexa Fluor 488‐conjugated donkey anti‐rabbit IgG from Abcam (Cat. # 150073), and Alexa Fluor 555‐conjugated phalloidin (A34055) and DAPI (Cat. # 28718‐90‐3) from Thermo Fisher Scientific.

### 2.2 Preparation of Ce6 nanoparticles (NPs)

PEG‐PLGA and Ce6 solutions were mixed in tetrahydrofuran (THF) at final concentrations of 0.5 and 0.15 mg/mL, respectively. Next, 1 mL of the mixture was injected quickly into 10 mL of ultrapure water under sonication followed by an additional 2 min of sonication. The contained THF was removed through steady nitrogen purging on a hot plate at ∼100°C, and other aggregates were removed by filtration through a porous membrane (pore diameter, 220 nm).

### 2.3 Optical characterization of Ce6 NPs

The shape and size of Ce6 NPs were assessed using transmission electron microscopy (TEM; using a Hitachi HT7700), and the NP hydrodynamic diameter was quantified using dynamic light scattering, Zeta potential and polydispersity index (PDI) were all determined using a Malvern Zetasizer Nano (ZS). The emission spectrum of Ce6 NPs was recorded by using a Hitachi F‐4500 fluorescence spectrometer with 400‐nm excitation wavelength, and the NP UV−vis absorption spectra were obtained on a Shimadzu UV‐2600 instrument. To detect ROS generation, the absorption change of ADMA at 259 nm was monitored. The Ce6 NPs and ADMA were mixed and diluted to 5 and 20 μg/mL with ultrapure water, the mixed solutions were irradiated with 650‐nm light from a light‐emitting diode (LED) at an intensity of 50 mW/cm^2^, and the absorption spectra were measured at 1‐min intervals. The RB solution served as a positive control for Ce6 NPs and was subjected to similar procedures with 520‐nm LED light being used for irradiation.

### 2.4 Mice

In this study, 4–6‐week‐old C57BL/6 strain mice were used, with their sex being selected at random. All experimental procedures performed on the animals were approved by the Southern University of Science and Technology Institutional Animal Care and Use Committee (SUSTC‐JY2019078).

### 2.5 Cell and cochlear explant cultures

HEI‐OC1 (House Ear Institute‐Organ of Corti 1) cells were cultured in high‐glucose Dulbecco's Modified Eagle's Medium (DMEM; Gibco Bethesda Research Laboratories [BRL]) containing 10% fetal bovine serum (FBS; Gibco BRL) without antibiotics under an atmosphere of 5% CO_2_ at 33°C. For cochlear explant culture, inner ears were dissected from sacrificed postnatal day 3 (P3) mice and placed in Hank's Balanced Salt Solution buffer, and then cochleae were dissected from the inner ears and adhered on 35‐mm dishes as reported previously.^16^ The cochlear explants were cultured in DMEM/F‐12 medium containing 1% FBS and 1.5 μg/mL ampicillin,^17^ under an atmosphere of 10% CO_2_ at 37°C overnight before PDT treatment.

### 2.6 PDT in vitro

PDT was applied to HEI‐OC1 cells and cochlear explants that were cultured overnight. In the case of HEI‐OC1 cells, Ce6 NPs of various concentrations were added onto cells cultured in 96‐well plates (at 3000 cells/well), and this was followed by irradiation with 650‐nm LED light at a dose of 50 mW/cm^2^ for 10 min. Cell viability was assessed after 24 h using a CCK‐8 cell counting kit (Dojindo Laboratories). Cochlear explants were treated with 3 μg/mL Ce6 NPs and irradiated with the same dose of light as that used for HEI‐OC1 cells. After culture for an additional 6 h to allow the PDT sufficient time to exert its effect on cells, the explants were fixed with 4% paraformaldehyde (PFA) for immunostaining.

### 2.7 Immunostaining

Inner ear specimens from mice or in vitro cochlear explants were immunostained after PDT treatment. Inner ears were isolated from the temporal bones and immersed in 4% PFA at 4°C with rotation overnight, and then decalcified in EDTA for 48 h. Subsequently, cochlear ducts were dissected out from the inner ears, and the modiolus was removed from the cochlea by using forceps. Next, the cochleae were incubated in Buffer 1 [phosphate buffered saline (PBS) mixed with 5% Bovine Serum Albumin (BSA) and 1% (v/v) Triton X‐100] for 1 h at room temperature for cell permeabilization, and then incubated overnight at 4°C with an anti‐myosin 7a primary antibody (1:500 dilution) in Buffer 2 [PBS with 5% BSA and 0.1% (v/v) Triton X‐100]. The specimens were washed three times with PBS and incubated with an appropriate secondary antibody (Alexa Fluor 488‐conjugated donkey anti‐rabbit IgG, 1:200) in Buffer 2 for 1 h in the dark at room temperature. In certain experiments, the stereocilia and nuclei of hair cells in horizontal semicircular canal (HSC) cristae were labeled, after secondary antibody staining, using Alexa Fluor 555‐conjugated phalloidin and DAPI, respectively.

### 2.8 FM1‐43 dye uptake

P4 mouse cochleae were adhered on 35‐mm dishes after dissection, cultured in DMEM/F‐12 medium overnight, and then treated with PDT: 5 μg/mL Ce6 NPs and 650‐nm LED irradiation at 50 mW/cm^2^ for 10 min. At 6 h after the treatment, FM1‐43 dissolved in Dimethyl Sulfoxide at 5 mmol/L was diluted 1:1000 in the culture medium and applied to the cultured cochleae. The cochleae were imaged using a confocal microscope after exposure to FM1‐43 for 3 min. The total depth of the confocal image stack was set at ∼8 μm to span over the hair buddle and the soma of vestibular hair cells.

### 2.9 ROS detection

The cellular ROS was detected by staining with H_2_DCFDA (Invitrogen). P4 mouse cochleae were adhered on 35‐mm dishes after dissection, cultured in DMEM/F‐12 medium overnight, and then treated with PDT: 5 μg/mL Ce6 NPs and 650‐nm LED irradiation at 50 mW/cm^2^ for 10 min. At 6 h after the treatment, all the cochleae were washed by PBS and then stained by 50 μM H_2_DCFDA for 10 min. ROS in HEI‐OC1 cells was detected similarly. The fluorescence images were taken by a confocal microscope.

### 2.10 PDT in vivo

Mice were anesthetized by intraperitoneally injecting a mixture of Zoletil^TM^ 50 (40 mg/kg body weight) and Dexdomitor (0.1 mg/kg body weight) in a volume of 0.04 mL/10 g body weight. All ear surgeries were performed unilaterally, with the ear on the other side serving as the non‐surgery control. The mice were placed on a heating pad to maintain the body temperature at ∼37°C during surgery. The postauricular area was depilated, the skin was sterilized with 75% alcohol, and all surgical instruments were sterilized using UV. A C‐shaped incision was made behind the right ear pinna under an operation microscope, and the cervical‐auricular muscles were separated by blunt dissection to expose the HSC. A polyimide transfer tube (ID: 0.005^’’^, OD: 0.0065^’’^; Nordson Medical; Lot No: 210302‐33) was connected to a polythene tube (inner and outer diameters, 0.28 and 0.61 mm, respectively; Smith Medical, Cat. # 263200), the end of which was connected using a 1‐mL syringe needle. A small hole was made using an insulin syringe needle (30G) on the HSC, and the polyimide transfer tube was inserted into the hole for perfusing the NPs. The fluid was delivered using a syringe pump controller (LongerPump, TJ‐2A) at a flow rate of 5 μL/min for 10 min. An optical bare fiber of 650‐nm wavelength directly targeted the hole on the HSC to trigger PDT after perfusion. The laser irradiation was set at a dose of 0.5 mW for 10 or 2 min. After the PDT treatment, the hole was sealed immediately with tissue glue and the skin was stitched. The entire surgical procedure lasted ∼30 min. To verify that Ce6 NPs were successfully delivered into the vestibular labyrinth through the HSC by using this approach, phalloidin was used as a tracer in a few initial experiments and administrated using the same method to label the hair bundles of vestibular hair cells, after which the vestibular organs were dissected out and examined for phalloidin labeling.

### 2.11 Vestibulo‐ocular reflex (VOR) test

The function of the semicircular canals of mice was evaluated using the VOR test. The VOR signal was recorded using a customized system named Mouse Vestibular Function Comprehensive Test System [Shenzhen Giant (Ju an) Technologies Co., Ltd, Cat. # GAT‐MVOR943]. Mice were mounted on a platform and their eye movements were recorded using a motion camera while the platform was rotated at various velocities and frequencies (rotation frequency: 0.5, 0.8, 1.6, or 3.2 Hz; uniform angular velocity: 40°/s). The duration of the rotation stimulus was >90 s. The eye‐movement amplitude was measured, and the corresponding gain value, defined by the ratio of averaged eye velocity to the platform velocity (40°/s), was calculated using a previously published algorithm.^18^, ^19^

### 2.12 Off‐vertical axis rotation (OVAR) assay

The OVAR assay was used to evaluate the function of otolith organs, as described previously.^20^ The OVAR system used in this study was manufactured based on the VOR system developed previously.^18^ The illumination and video‐detection devices were installed on an octagonal motion platform (diameter, 30 cm), as well as on the mouse holder in the platform center. The platform was driven by a stepper motor, allowing sinusoidal rotation motion according to the designed rotation modes. The illumination for the video recording was composed of two near‐infrared (NIR) LED lamps (940 nm, Chundaxin®, China). A sports camera (MI®, China) was used to detect the NIR images of the mouse eyes on each side, which were reflected by two hot mirrors placed in front of the mouse eyes. To detect eye movements, the mouse was fixed on the platform center and a constant velocity of 30°/s, 50°/s, or 80°/s was used, and the entire chamber was tilted 17° with respect to the ground.^21^ The amplitudes of the eye movements were quantified.

### 2.13 Auditory brainstem response (ABR)

Mice were anesthetized in a manner similar to the preparation for surgery, and the ABR test was performed using a Tucker‐Davis Technologies RZ6/BioSigRZ system (Tucker‐Davis Technologies, USA) in a sound‐proof room. The intensity of the tone‐burst stimulus was set from 90 to 10 dB sound pressure level at various frequencies ranging from 4 to 32 kHz. During the test, mice were kept warm on a heating pad.

### 2.14 Cell counting

Hair cells from both the cochlea and the utricle that displayed normal nuclei and were clearly labeled with anti‐myosin 7a were considered live cells. The cochlea and utricle were imaged using a Zeiss Microscope, and live cells were counted using ImageJ software. For the cochlea, average numbers of hair cells per 100 μm in the middle portion of each cochlear explant (∼50% from the apical turn) were selected for counting.

### 2.15 Imaging and statistical analysis

All the images were acquired by a Zeiss LSM800 confocal microscope. Myosin7a images of HSC were obtained using the same setting of the microscope. Averaged myosin7a fluorescence intensity of three representative areas in each HSC (50 × 50 μm^2^) represented the hair cell viability. The fluorescence intensity was measured by the ImageJ software. All data are shown as means ± standard deviation. Unpaired Student's *t* test was used for statistical analysis; *p* < 0.05 was considered statistically significant.

## 3 RESULTS

### 3.1 Generation and characterization of Ce6 NPs

We selected the second‐generation photosensitizer Ce6 for PDT because of its high singlet‐oxygen quantum yield, low toxicity, and rapid clearance, and additionally because the NIR florescence of Ce6 allows deep penetration in live tissues and is compatible for florescence imaging.^22^, ^23^, ^24^, ^25^ Ce6 is also currently under clinical trials for the treatment of various cancers.^26^ In the present study, Ce6 was packed in NPs prepared using PLGA as the shell and PEG as the shell coating,^27^ and the NP packing substantially increased the solubility of hydrophobic Ce6. PLGA has been widely used in nanomedicine, including in hearing research, primarily due to its high biodegradability and biocompatibility.^28^, ^29^, ^30^

**FIGURE 1:**
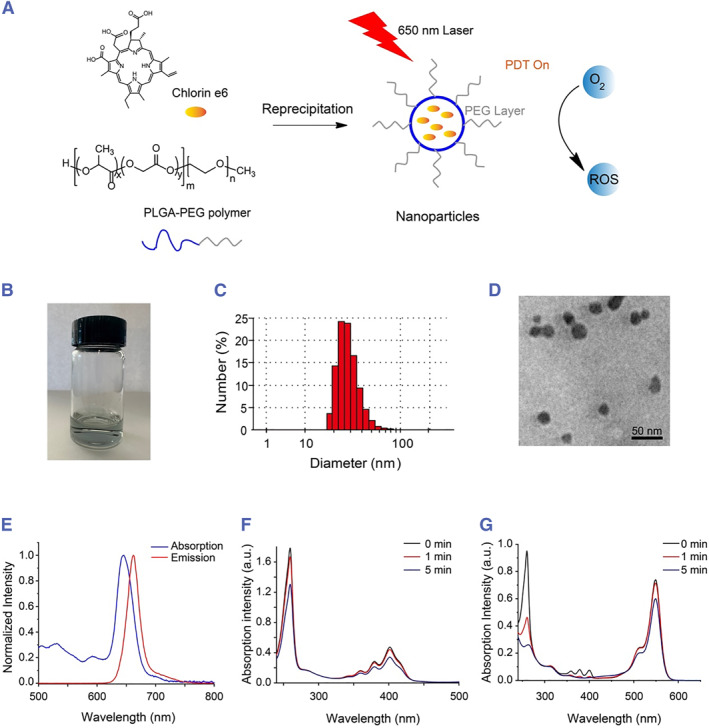
Preparation and optical characterization of Ce6 NPs. (A) Schematic of Ce6 NP preparation and activation. (B) Image of Ce6 NP solution. (C) Distribution of Ce6 NP diameters measured using DLS. (D) TEM micrograph of Ce6 NPs. (E) Normalized absorption and emission spectra of Ce6 NPs. (F–G) Time series of ADMA absorption spectrum upon activation of either Ce6 NPs by 650‐nm light (F) or rose bengal by 520‐nm light (G). ADMA, 9,10‐anthracenediyl‐bis (methylene) dimalonic acid; DLS, dynamic light scattering; NPs, nanoparticles; TEM, transmission electron microscopy.

The preparation of Ce6‐doped PEG‐PLGA NPs (Ce6 NPs) and the principle of PDT are shown in Figure 1A. Ce6 exhibits strong absorption at ∼650 nm and can be excited by 650‐nm light to produce ROS. The Ce6 NPs were generated using the reprecipitation method as reported previously,^31^, ^32^ and the resulting NP solution appeared transparent (Figure 1A,B). The average hydrodynamic diameter of the Ce6 NPs was ∼20–30 nm (Figure 1C,D), the zeta potential −25 mV, and the PDI 0.512. The stability of the NPs was evaluated by the size change on the day0, day7 and day30, respectively, which was stable during the first month after NPs preparation (Figure S1). The NPs also exhibited peak absorption at ∼650 nm and emission at ∼670 nm as expected (Figure 1E). Moreover, ROS generation by the NPs was confirmed using ADMA, a chemical ROS reporter.^33^ The absorption of ADMA at 259 nm is quenched by ROS due to photodegradation,^34^, ^35^ and, accordingly, the ADMA absorption intensity at 259 nm was considerably diminished upon 650‐nm‐light excitation of Ce6 NPs (Figure 1F), which verified the ROS‐generating ability of the NPs. The validity of ADMA as a chemical ROS reporter was confirmed by exciting the commonly used photosensitizer RB (Figure 1G).^36^

### 3.2 PDT induced cell death in HEI‐OC1 cell line and cochlear explants in vitro

**FIGURE 2:**
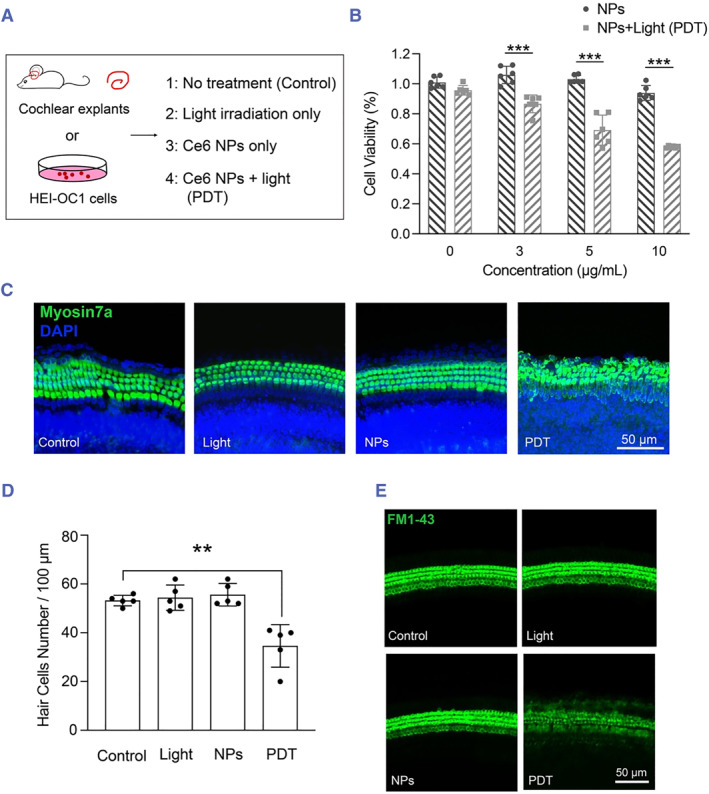
PDT effect on HEI‐OC1 cells and cochlear explants. (A) Experimental setup. Cells or explants were not treated (control) or treated with 650‐nm light for 10 min (light irradiation only), Ce6 NPs only, or Ce6 NPs + light irradiation (PDT). (B) Number of live cells (quantified using the CCK‐8 assay) after PDT treatment with different concentrations of Ce6 NPs with or without light irradiation; *n* = 6 biological replicates. (C) Staining of myosin 7a antibody (green; labeling of hair‐cell body) and DAPI (blue; labeling of nucleus) in the cochlea in control and experimental groups. (D) Number of myosin 7a‐positive hair cells per 100 μm of cochlear explants shown in panel C; *n* = 5 cochlear explants. (E) Images of cochlear explants upon FM1‐43 exposure. **p < 0.01, ***p < 0.001. NPs, nanoparticles; PDT, photodynamic therapy.

Because little is known regarding PDT efficacy in the case of hair cells, we first assessed how PDT affects hair cells in vitro before performing more complex and time‐consuming in vivo experiments. In one set of experiments, we tested the PDT effect on the viability of the HEI‐OC1 cell line, which is derived from long‐term cultures of cochleae and expresses several specific molecular markers of sensory hair cells and supporting cells. The experimental setup is shown in Figure 2A. Considering previous work involving Ce6‐based PDT on cancer cells,^37^ we selected 3, 5, and 10 μg/mL as the Ce6 NP concentrations for our PDT assay. At 24 h after treatment, which provided sufficient time for the PDT to exert its effect, the viability of HEI‐OC1 cells was decreased in a Ce6 dose‐dependent manner (Figure 2B). Notably, PDT applied with 10 μg/mL Ce6 NPs damaged nearly 50% of the cells. Conversely, no cytotoxicity was detected with either Ce6 NPs or 650‐nm irradiation alone.

In another set of experiments, we tested the PDT effect on hair cells of mouse cochlear explants. At 6 h after PDT, the soma of hair cells appeared disintegrated, and the number of hair cells per 100 μm of cochlear length decreased significantly (Figure 2C,D). By contrast, Ce6 NPs or light irradiation alone failed to cause cell death (Figure 2C,D). We also investigated the PDT effect on hair‐cell function by performing the FM1‐43 dye‐uptake assay, which provides a functional readout of the mechanoelectrical transduction (MET) channel.^38^ Cochlear explants from P3 mice were exposed to FM1‐43 dye for 3 min, a short exposure time that ensures FM1‐43 uptake primarily by rapid entry through the MET channel rather than by slow endocytosis.^39^, ^40^ While robust FM1‐43 uptake was observed in cochlear inner and outer hair cells treated with the NPs or light irradiation alone, the dye uptake was markedly diminished in PDT‐treated hair cells (Figure 2E). In addition, the PDT increased the intracellular ROS in both the cochlear explants and HEI‐OC1 cells as expected (Figure S2). Collectively, our results suggested that the PDT dose used here effectively damaged hair cells, but Ce6 NPs or 650‐nm light alone produced no cytotoxicity.

### 3.3 PDT attenuated HSC function in vivo

The semicircular canals, a major diseased site in peripheral vertigo, comprise three parts: the horizontal, anterior, and posterior canal (HSC, ASC, and PSC) segments. Although the PSC and HSC are the major parts involved in BPPV and the ASC is seldom associated with BPPV, all three parts are involved in other disease‐related vertigo to a certain extent.^41^, ^42^ Thus, all three segments represent targets in vertigo treatment. After establishing the PDT effect on HEI‐OC1 cells and on hair cells in cochlear explants, we used a mouse model to investigate the PDT effect on semicircular canals in vivo. Because the functional assay for the semicircular canal is commonly designed for the HSC but not the ASC and PSC in the mouse, we focused on the PDT effect on the HSC in this study.

**FIGURE 3:**
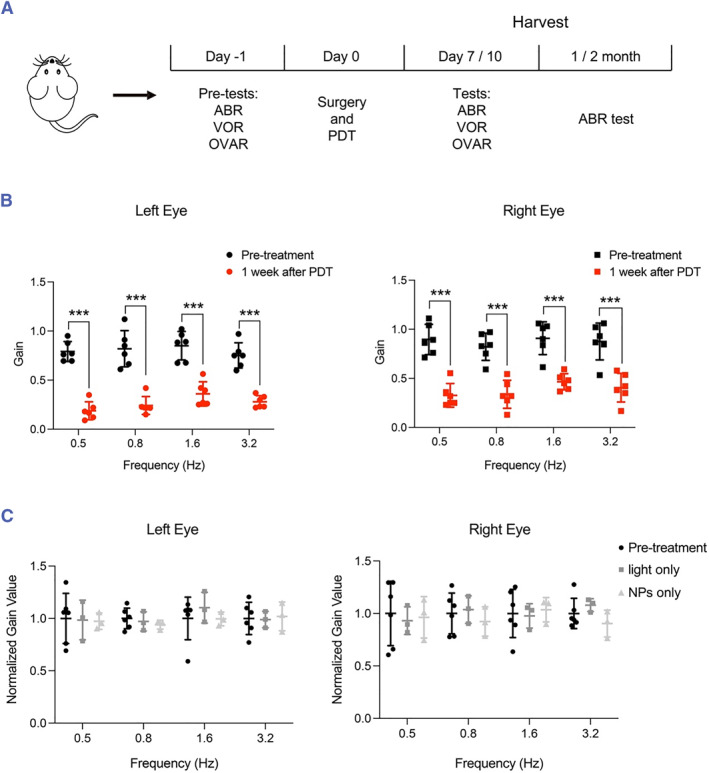
Effect of PDT on HSC in mice. (A) Experimental design of in vivo PDT assay. VOR, OVAR, and ABR assays were used to evaluate the function of HSC, utricle, and cochlea, respectively. (B) Gain value from VOR tests of contralateral (left) and ipsilateral (right) eyes of mice at 1 week after PDT treatment. Pre‐treatment: before PDT; *n* = 6. (C) Normalized gain value of eye movement in pre‐treatment (*n* = 6), light irradiation only (*n* = 3), and Ce6 NP treatment only (*n* = 3). **p < 0.01, ***p < 0.001 versus pre‐treatment group. ABR, auditory brainstem response; HSC, horizontal semicircular canal; NPs, nanoparticles; OVAR, Off‐vertical axis rotation; PDT, photodynamic therapy; VOR, vestibulo‐ocular reflex.

**FIGURE 4:**
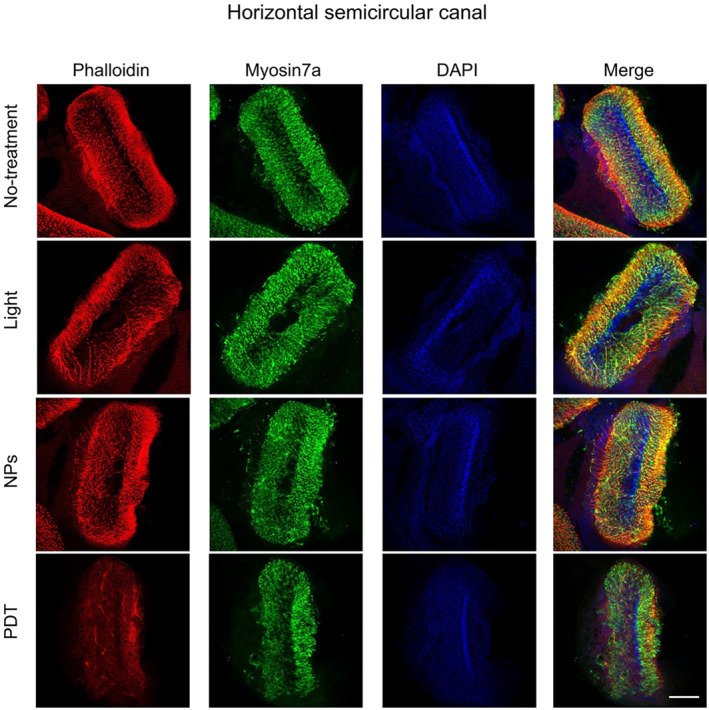
Hair‐cell viability in HSC after PDT. Labeling of cristae ampulla of HSC with phalloidin (red), myosin 7a antibody (green), and DAPI (blue) at 1 week after treatment: no‐treatment, light irradiation only, Ce6 NPs only, or PDT. Scale bar: 100 μm. HSC, horizontal semicircular canal; NPs, nanoparticles; PDT, photodynamic therapy.

Mice were first subjected to PDT treatment of the HSC and allowed to recover from the surgery for a week (Figure 3A), and then the PDT effect on HSC function was assessed using the VOR test. Notably, the gain value of the contralateral eyes in the VOR test was significantly decreased in all four frequency modes tested, which clearly indicated PDT‐induced attenuation of HSC function (Figure 3B, left). Similar results were obtained in the ipsilateral eye movement (Figure 3B, right). The weakened bilateral eye movement after PDT treatment of unilateral HSC is expected because the unilateral HSC controls both the contralateral lateral rectus and ipsilateral medial rectus of the extraocular muscles.^43^ Importantly, Ce6 NPs or 650‐nm light alone did not affect the bilateral eyes in VOR tests (Figure 3C). Moreover, viability analysis of hair cells in cristae ampulla of HSC (Figure 4) revealed that whereas irradiation or Ce6 NPs alone produced no effect on hair‐cell viability or morphology, PDT treatment markedly damaged hair cells, as indicated by reduced actin and myosin 7a labeling (Figure S3). These results agree with the functional data (Figure 3).

### 3.4 High‐dose PDT attenuated function of otolith organs in vivo

**FIGURE 5:**
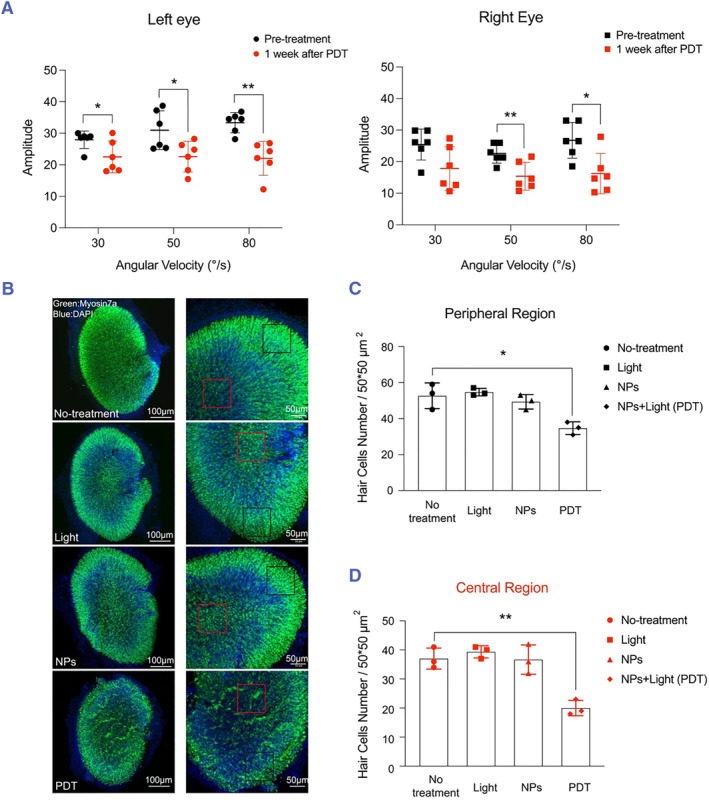
Effect of PDT on the utricle. (A) Amplitude of contralateral (left) and ipsilateral (right) eye movements measured using Off‐vertical axis rotation (OVAR) assays pre‐treatment and 1 week after PDT. (B) Immunolabeling of myosin 7a (green; hair‐cell body) and DAPI staining (blue; nucleus) of utricle in no‐treatment, light‐irradiation, Ce6 NP, and 1‐week post‐PDT groups. Right images: magnification of corresponding left images: black box, example of peripheral region; red box, example of central region. (C–D) Summary data of hair‐cell numbers from peripheral (C) and central (D) regions of utricle in Panel B. *p < 0.05, **p < 0.01; *n* = 3 mice for panels C and D. NPs, nanoparticles; OVAR, Off‐vertical axis rotation; PDT, photodynamic therapy.

Because otolith organs—the utricle and saccule—are anatomically adjacent to the HSC, we tested whether the PDT dose used in our experiments also damaged the otolith organs. We examined the effect of PDT on the otolith organs using the OVAR assay, which provides a functional readout of both the utricle and the saccule, although the assay is predominantly used to assess the utricle.^21^ OVAR assays performed 1 week after the PDT on the HSC revealed that in PDT‐treated mice, the amplitude of the contralateral eye movement was significantly decreased in all angular velocity modes, and the amplitude of the ipsilateral eye movement was markedly decreased under the angular velocities of 50°/s and 80°/s (Figure 5A).

To corroborate the functional data, we also examined the effect of PDT on utricular hair cells. Utricular hair cells showed abnormal aggregation and a decreased myosin 7a signal (green) following PDT, which reflected hair‐cell damage (Figure 5B). In contrast, hair cells in the control groups appeared normal (Figure 5B). Furthermore, we quantified the effect of PDT on the number of hair cells. Because utricular hair‐cell distribution is not homogenous, we counted cells within randomly selected areas (boxes in Figure 5B) in the peripheral and central regions of the utricle, which revealed a significant reduction in the average number of hair cells per unitary area of the peripheral and central regions after PDT treatment (Figure 5C,D). These results showed PDT‐induced damage of utricular hair cells, consistent with the functional assay results in Figure 5A.

Notably, the otolith organs are also regarded as the diseased site in non‐spinning vertigo,^44^ and certain patients with vestibular disorders such as BPPV and MD were reported to present abnormalities in the otolith organs.^45^, ^46^ In one study, a patient with non‐spinning vertigo symptoms was diagnosed with isolated otolith dysfunction and was treated with vestibule plugging surgery to obstruct the vestibular window and thereby abolish the function of otolith organs.^47^ Thus, attenuating the function of otolith organs by PDT is of clinical significance in the treatment nonspinning vertigo.

### 3.5 PDT did not affect hearing function

**FIGURE 6:**
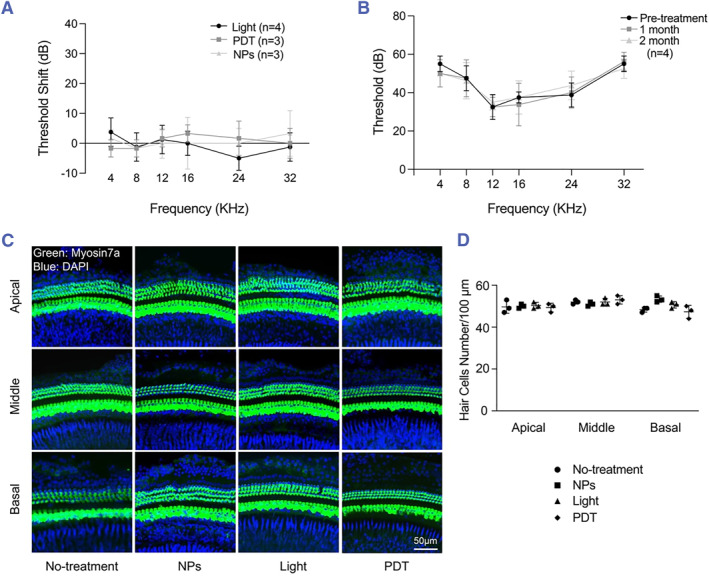
Effect of PDT on mouse cochlea. (A) ABR threshold shift relative to pre‐treatment in mice, and treated with light irradiation, Ce6 NPs, and PDT. (B) ABR threshold before, 1 month after, and 2 months after Ce6 NP treatment alone. (C) Labeling of apical, middle, and basal turns of cochleae with myosin 7a antibody and DAPI. (D) Number of myosin 7a‐positive hair cells per 100 μm in apical, middle, and basal cochlear turns in Panel C; *n* = 3 cochleae. ABR, auditory brainstem response; NPs, nanoparticles; PDT, photodynamic therapy.

We reasoned that in our experimental model, minimal damage to the cochlea would occur because the irradiation site on the HSC is far from the cochlea. To verify this, we assessed the hearing function of PDT‐treated mice using ABR measurements, which revealed, as expected, that PDT caused a minimal shift in the ABR threshold (Figure 6A). Furthermore, we examined the long‐term effect of Ce6 NPs alone on hearing function and measured no marked effect of the NPs on the ABR threshold at one or 2 months after the treatment (Figure 6B), which suggested that the potential diffusion of Ce6 NPs into the inner ear labyrinth does not adversely affect hearing function in the long term. Moreover, we observed no significant loss or abnormal morphology of hair cells in the cochlea after PDT (Figure 6C,D). These results suggested that the vestibular‐targeted PDT treatment in our study exerted no or minimal effect on hearing function.

### 3.6 Low‐dose PDT specifically weakened HSC function without affecting otolith organs

**FIGURE 7:**
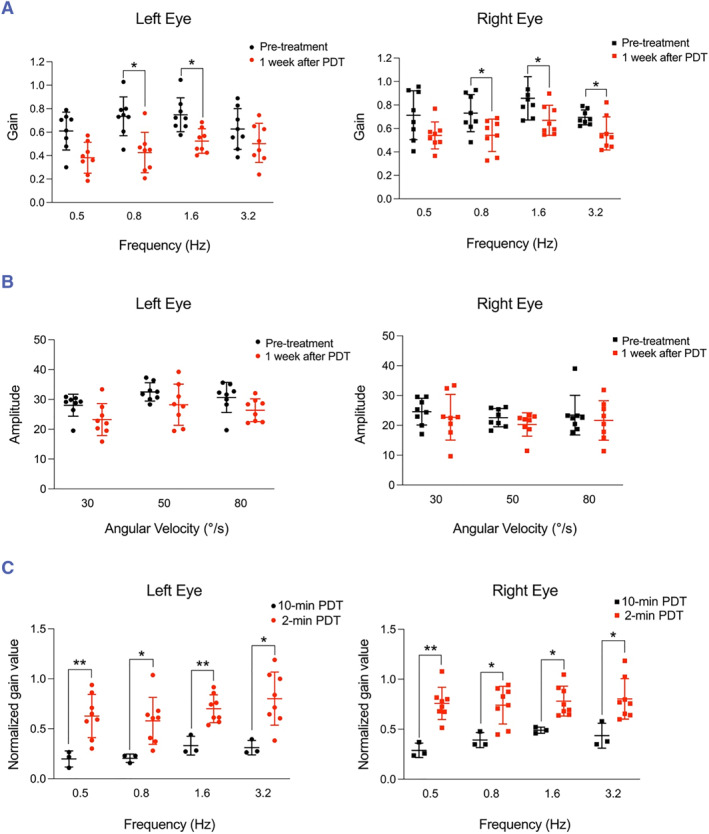
Reduced PDT dose induces focused cell damage in HSC. (A) Gain value of contralateral (left) and ipsilateral (right) eye movement of mice in VOR tests at 7 days after 2‐min PDT; *n* = 8. (B) Amplitude of contralateral (left) and ipsilateral (right) eye movement of mice in OVAR tests at 7 days after 2‐min PDT; *n* = 8. (C) Comparison of VOR test results between 2‐ and 10‐min PDT after normalization. *p < 0.05, **p < 0.01. HSC, horizontal semicircular canal; OVAR, Off‐vertical axis rotation; PDT, photodynamic therapy; VOR, vestibulo‐ocular reflex.

The results are shown in Figure 5 suggested that the PDT dose used in our experiments induced concurrent damage to the otolith organs. Thus, we hypothesized that a reduced PDT dose could induce a more focused damage of the HSC without affecting the adjacent otolith organs. To test this, we reduced the light‐irradiation time from 10 to 2 min and examined the function of both the HSC and otolith organs. Notably, the gain value of the contralateral eye movement at 0.8 and 1.6 Hz was significantly decreased 1 week after PDT, and the gain value of the ipsilateral eye movement at 0.8, 1.6, and 3.2 Hz was also significantly decreased (Figure 7A). These results suggested that even the reduced PDT dose successfully elicited cell damage in the HSC. Importantly, the results of the OVAR assay showed no significant change at 1 week after PDT (Figure 7B), which indicated that the function of the otolith organs remained intact when the reduced dose of PDT was used. We also analyzed the difference in the effect on HSC function of PDT applied with 10‐ and 2‐min light irradiation: The normalized values of the contralateral and ipsilateral eye movements at all frequency modes in the 2‐min PDT group were significantly increased, which indicated diminished HSC injury relative to that after 10‐min PDT (Figure 7C). This reduced impairment of HSC function after the low‐dose PDT still holds considerable potential for clinical application; a previous clinical study reported that patients who were administered ITGI several times to control the symptoms of severe vertigo showed a reduction of the VOR gain value, and ∼17.8% of the VOR gain reduction in the HSC was counted as the endpoint of the treatment.^48^ The percentage reduction of the VOR gain in our low‐dose PDT experiment is comparable to this value.

### 3.7 Light irradiation through bone can trigger effective PDT in vivo

**FIGURE 8:**
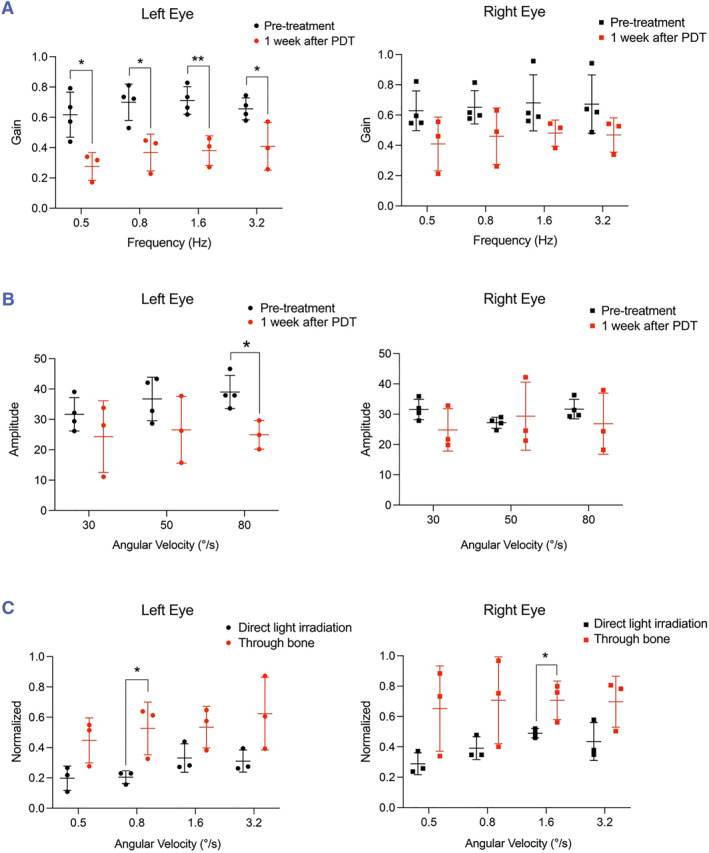
PDT performed using light irradiation through bone results in reduction of VOR. (A) Gain value from VOR tests of left and right eyes of mice at 1 week after PDT activated by light irradiation through bone. Pre‐treatment: before PDT, *n* = 4; 1 week after PDT, *n* = 3. (B) Amplitude from OVAR tests of left and right eyes of mice at 1 week after PDT activated by light irradiation through bone. (C) Normalized value from VOR tests for PDT performed using light irradiation either directly through a hole on HSC or through bone. *p < 0.05, **p < 0.01. (better to give the largest of *p* values). HSC, horizontal semicircular canal; OVAR, Off‐vertical axis rotation; PDT, photodynamic therapy; VOR, vestibulo‐ocular reflex.

In all the in vivo PDT experiments described above, light was delivered by placing the optical fiber on top of a small hole drilled on the HSC. We next examined the feasibility of PDT performed using light irradiation through the bone of the vestibular labyrinth. We reasoned that light irradiation through the bone would enable us to trigger PDT at any location on the semicircular canals without drilling a hole, particularly at the commonly diseased site in BPPV—the PSC—where drilling a hole is surgically challenging. The drug‐delivery method used here was the same as in the experiment described earlier, but we positioned the optical fiber far from the hole and near the ampulla of the HSC to trigger PDT through the bone of the vestibular labyrinth. At 1 week after 10‐min light irradiation through the bone for PDT (light power, 0.5 mW), the gain value of the left eye was significantly decreased under all rotation modes (Figure 8A, left). Moreover, although the difference measured in the case of the right eye was not statistically significant, the average gain was diminished after the PDT (Figure 8A, right). The OVAR test results further showed that the function of the otolith organs was mostly unchanged (Figure 8B). Thus, we maintained the normal function of the otolith organs and locally abated the function of HSC by means of PDT triggered by light irradiation through bone. Lastly, the normalized value from the VOR test (Figure 8C) after PDT with light irradiation through the bone showed that a portion was significantly increased as compared with the test described above; this was as expected and presumably because the light attenuation after bone penetration led to lower PDT dosage. These results showed the feasibility of using light irradiation through bone for PDT.

## 4 DISCUSSION

### 4.1 Current developments and limitations in vertigo treatment

Approximately 10%–35% of patients with vertigo experience body disequilibrium, frequent falls, nausea, and vomiting, which seriously affect the daily activities of the patients,^49^, ^50^ and 80% of all vertigo cases are due to dysfunction of the peripheral vestibular apparatus.^51^ Although nonsurgical procedures and medication are effective for mild symptoms, severe cases might require surgery or destructive treatments such as endolymphatic sac surgery, semicircular canal occlusion, ampullary neurotomy, and ITGI.

Endolymphatic sac decompression is performed to release the pressure of the endolymph and thereby relieve the vertigo symptoms of patients with MD, resulting from endolymphatic hydrops.^52^ However, endolymphatic hydrops might not account for all the vertigo experienced in MD. Therefore, endolymphatic sac decompression shows low effectiveness and exhibits little evidence‐based effect on the vertigo in MD.^52^, ^53^, ^54^ Moreover, although endolymphatic sac decompression surgery successfully relieves symptoms in certain cases, the surgery might produce an unpredictable side effect on hearing function because the cochlea would inevitably be involved in the surgery and could potentially be damaged.^55^

Transection of the ampullary nerve and semicircular canal occlusion are the primary surgery options for treating intractable BPPV.^56^ Transection of the ampullary nerve, also called ampullary neurotomy, is a method used to cut off the ampullary nerve to disrupt the innervation of semicircular canals. However, ampullary neurotomy can lead to severe complications such as sensorineural hearing loss and facial paralysis.^8^ In semicircular canal occlusion, the aim is to obstruct the liquid space between the tops of the canals to inhibit the activation of hair cells.^57^ Semicircular canal occlusion has been reported to effectively relieve vertigo, but patients who have undergone the surgery have been found to exhibit a short period of dysfunction of their hearing and vestibular system and in certain cases to show persistent hearing impairment, such as due to perilymph leakage and local inflammation.^8^ These limitations make it crucial to develop a precise method to damage a single semicircular canal.

Another therapy for refractory vertigo is ITGI, which is regarded as the most effective treatment for severe vertigo.^58^ The efficiency of ITGI is based on the ototoxicity of gentamicin leading to direct damage of vestibular type 1 hair cells and dark cells from the labyrinth.^9^ However, ITGI shows a detrimental effect on the adjacent cochlea and would consequently impair hearing function.^59^ A previous study reported that ∼25% of patients exhibited impaired hearing after ITGI, with ∼20 dB pure tone average increase.^60^ Furthermore, the hair cells of the utricle and saccule were found to be damaged after ITGI, which revealed limited specificity for targeting a single vestibular organ.^61^

### 4.2 Improved methods based on PDT in this study

Because ITGI and surgical treatments are highly invasive and can lead to complications such as unpredictable hearing loss (discussed above), we used the principle of PDT to achieve precise damage of vestibular hair cells of the HSC in this study. We irradiated the targeted area with 650‐nm light to excite Ce6 NPs after their delivery to the targeted area. For light irradiation, a bare light fiber targeted a hole on the HSC (the same used for Ce6 NPs delivery) in most of our experiments (Figures 3, 4, 5, 6, 7). Our functional and immunostaining assay results showed attenuated function and hair‐cell injury of both the semicircular canals and otolith organs. Notably, the PDT exerted no effect on hearing function or cochlear hair‐cell viability. These results suggest the therapeutic potential of PDT for both common spinning vertigo and the less‐common non‐spinning vertigo resulting from dysfunctional semicircular canals and otolith organs. To specifically attenuate the function of semicircular canals to treat common spinning vertigo, we reduced the PDT dose, and we found that the low‐dose PDT induced precise injury of the semicircular canals without affecting the otolith organs (Figure 7). Furthermore, to increase the versatility of our approach, we modified the light‐irradiation method to irradiate the targeted area through bone, and the results of our functional analysis indicated that the delivered Ce6 NPs could be excited within the region of the semicircular canal even with the attenuation of light passing through the bone.

Due to the hydrophobic nature of the photosensitizer Ce6, we packed Ce6 with a biodegradable polymer, PEG‐PLGA, as the shell to assemble NPs to improve its water solubility and verified its safety.^62^ The NP‐based method of delivery is reported to control drug release, enhance cell uptake, and minimize side effects.^63^, ^64^ PLGA‐NPs have previously demonstrated improved distribution in the inner ear after delivery through the round window membrane.^65^ Furthermore, the use of PLGA‐based NPs modified the surface PEG decoration and thus promoted drug delivery to outer hair cells as a result of improved hydrophilicity.^66^ In this study, we also employed local drug delivery through the HSC, which can maximally deliver Ce6 NPs to the vestibular system. As shown in Figure 4, directly delivering phalloidin into the inner ear through the HSC led to successful labeling of hair cells, which verified the efficiency of this local drug‐delivery approach. However, the Ce6 NPs used in this study showed little specificity for vestibular hair cells after delivery into the inner ear, which could cause side effects on other nontargeted cells when PDT is triggered. The current approach has demonstrated a certain level of specificity,^66^ and with further technical improvement, such as an antibody coating strategy, the specificity to HCs can be foreseeably elevated. Additionally, other formation of nanosystems such as biomimetic nano@microgels, which have been verified to improve drug release and accumulation in the target area that could be considered as a potential method for drug delivery.^67^

### 4.3 Potential approaches based on PDT

Regarding the surgical method used in this study, we first exposed the HSC and drilled a hole on it to deliver the PDT drug. However, exposing the HSC for PDT would require passing through the mastoid process if this surgery is to be applied clinically to humans. A transmastoid approach for canal plugging in the treatment of semicircular canal dehiscence has been used for several years,^68^, ^69^ and this could serve as a reference in potential PDT applications. The HSC could be exposed using the transmastoid approach and then the drug could be delivered through a round or oval window, after which the laser can be directly targeted on the bone of the HSC for PDT activation. Furthermore, endoscopic stapes surgery is commonly used for otosclerosis treatment, which involves operation on the footplate of stapes placed on the oval window^70^, ^71^; this provides a minimally invasive approach to deliver the drug and laser into the vestibule through the middle ear by endoscopy. However, safe and minimally invasive surgical methods warrant further investigation, including the PDT drug‐delivery and light‐irradiation methods for future application in the clinic.

In summary, we have developed a novel method for attenuating the function of the vestibular organs or a single vestibular sub‐organ by using the principle of PDT. Our method shows increased spatial selectivity and safety in vertigo therapy compared with ITGI and occlusion surgery on semicircular canals. Although the lack of a mouse vertigo model limits further direct examination of the therapeutic efficiency of our PDT‐based approach for the disease, our study provides an animal experimental basis for the potential development of innovative vertigo treatment in the clinic.

## AUTHOR CONTRIBUTIONS

Conceptualization, Yingkun Yang, Pingbo Huang, and Fangyi Chen; methodology, Yingkun Yang, Pingbo Huang, and Fangyi Chen; experimental investigation, Yingkun Yang, Tong Zhao, and Feixue Mi; formal analysis, Yingkun Yang; writing original draft, Yingkun Yang and Pingbo Huang; editing and review, Pingbo Huang, Fangyi Chen and Hongzhe Li; supervision, Pingbo Huang and Fangyi Chen; funding acquisition, Fangyi Chen and Pingbo Huang.

## CONFLICT OF INTEREST STATEMENT

Fangyi Chen and Yingkun Yang are inventors of a filed Chinese patent (No. 202411168373X) on using PDT to impair vestibular functions. All authors declare that there are no competing interests.

## ETHICS STATEMENT

The study was approved by the Southern University of Science and Technology Institutional Animal Care and Use Committee (SUSTC‐JY2019078). Animal care and use conform to the guidelines of the Chinese Guide for the Care and Use of Laboratory Animals.

## Supporting information

Supporting Information S1
