# Supplementary material for: Photodynamic therapy‐induced precise attenuation of light‐targeted semicircular canals for treating intractable vertigo
Source: Smart Med. 2024 Oct 19;3(4):e20230044. doi: 10.1002/SMMD.20230044 (PMC11669792; doi:10.1002/SMMD.20230044)
Supplement: Supplementary file 1 — Supporting Information S1 [file SMMD-3-e20230044-s001.docx]

**Supplementary Data**

**Photodynamic therapy-induced**

**precise attenuation of light-targeted semicircular canals for treating intractable vertigo**

Yingkun Yang^1,2,3^, Tong Zhao^1^, Feixue Mi^1^, Hongzhe Li^6^, Pingbo Huang*^2,4,5^, and Fangyi Chen*^1^

^1^Department of Biomedical Engineering, Southern University of Science and Technology, Shenzhen, Guangdong, China.

^2^Division of Life Science, Hong Kong University of Science and Technology, Hong Kong, China.

^3^Department of Otolaryngology-Head and Neck Surgery, Stanford University, CA, United States.

^4^State Key Laboratory of Molecular Neuroscience, Hong Kong University of Science and Technology, Hong Kong, China.

^5^Hong Kong Branch of Guangdong Southern Marine Science and Engineering Laboratory (Guangzhou), Hong Kong University of Science and Technology, Hong Kong, China.

^6^VA Loma Linda Healthcare System, Loma Linda, CA, United States.

* To whom correspondence should be addressed: [bohuangp@ust.hk](mailto:bohuangp@ust.hk) and [chenfy@sustech.edu.cn](mailto:chenfy@sustech.edu.cn)

**Supplementary Figure 1. The size distribution of the NPs on Day 0, Day 7, and Day 30 after preparation.**


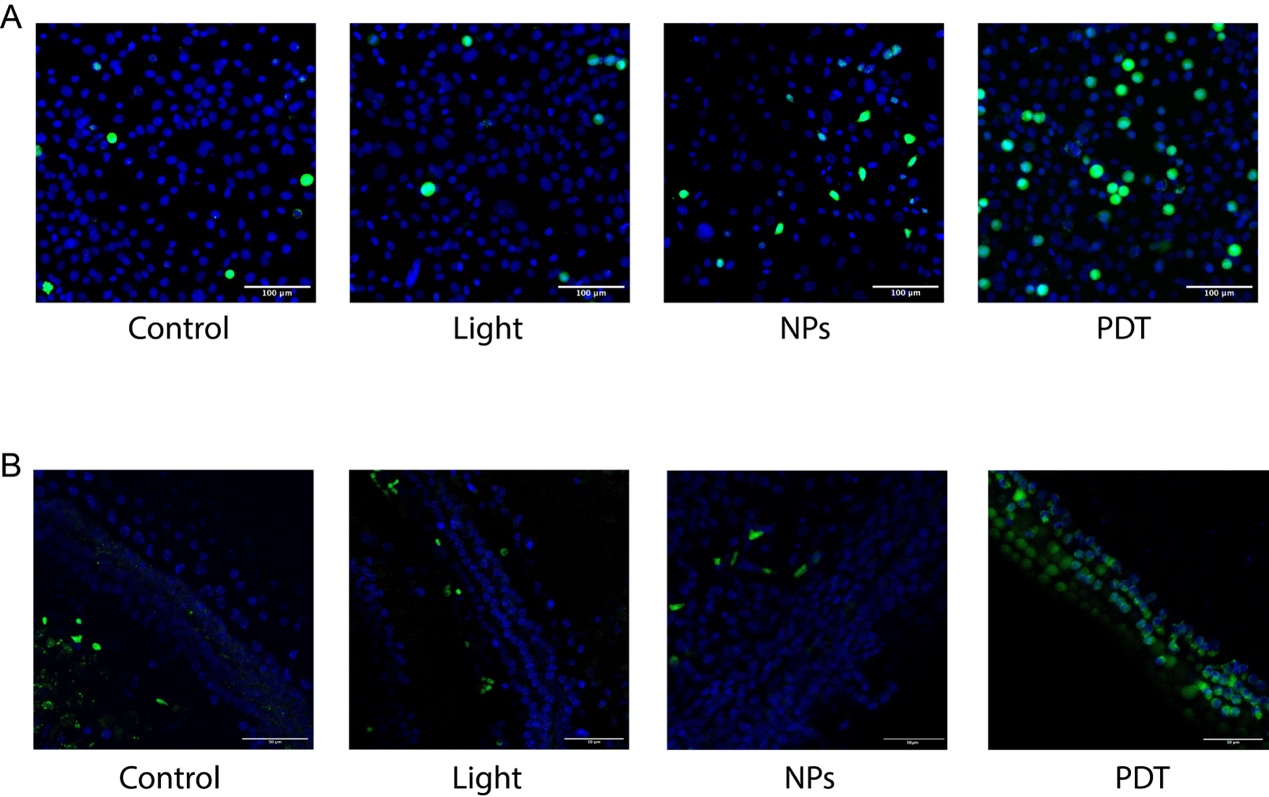


**Supplementary Figure 2. The production of ROS by PDT.** A. Representative fluorescence images of HEI-OCI cells that stained with H_2_DCFDA. Scale bar: 100 μm. B. Representative fluorescence images of cochlear explants that stained with H_2_DCFDA.Scale bar: 50μm. (Green: H_2_DCFDA; Blue: DAPI)


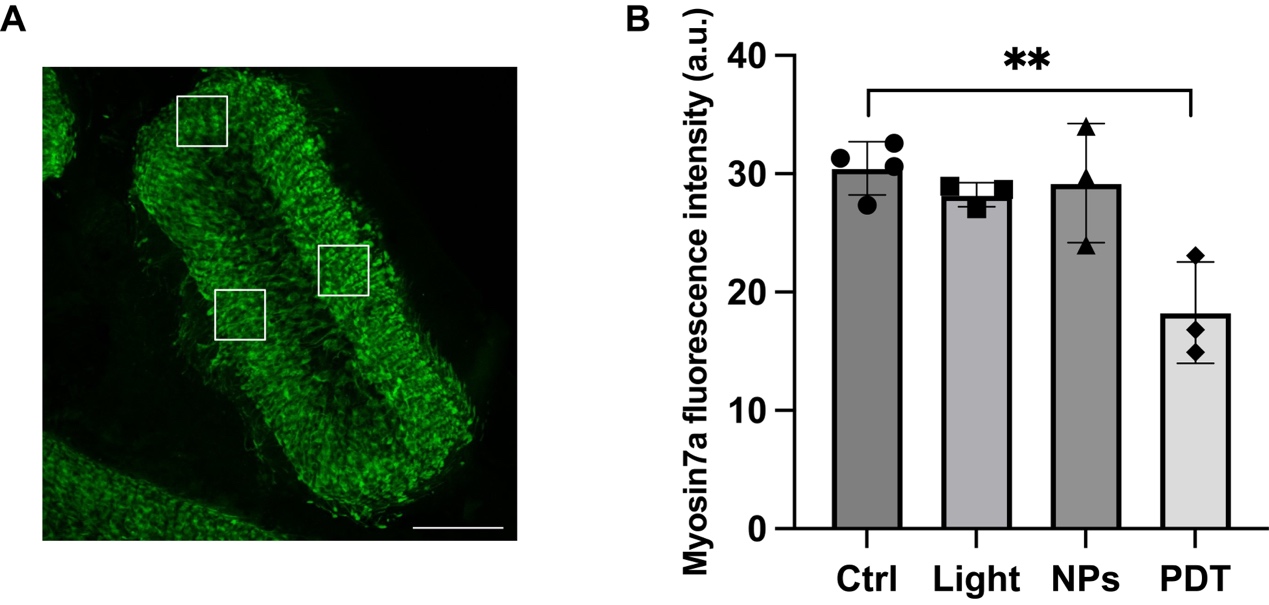


**Supplementary Figure 3. Quantified myosin7a fluorescence intensity in the HSC.** A. Three representative areas in the HSC, scale bar: 100 μm. B. Summary of fluorescence intensity of myosin 7a in the control, light irradiation, NPs delivery, and PDT groups in the *in vivo* assay. **, *p* = 0.0042.


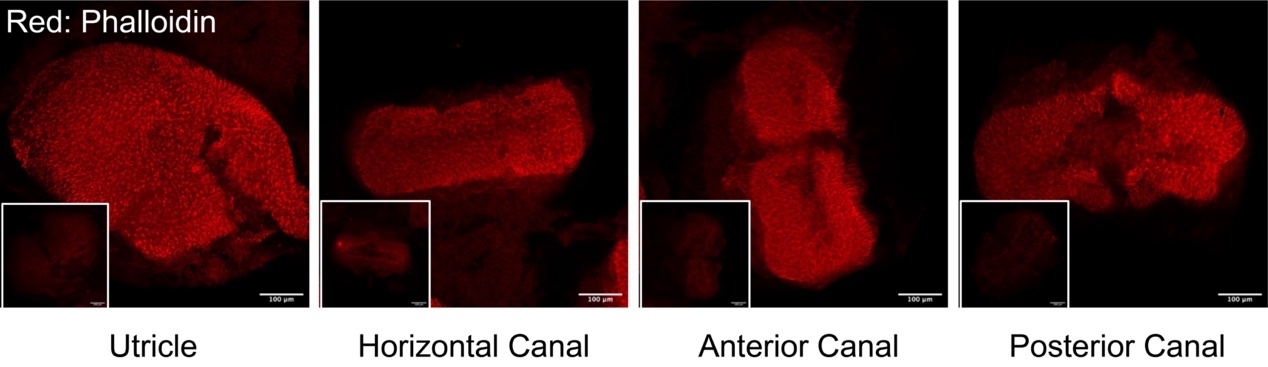


**Supplementary Figure 4.** **Vestibular organs after phalloidin delivery through hole on HSC.** Fluorescence images of vestibular organs: utricle, HSC, ASC, and PSC. Hair cells were labeled with phalloidin. Insets, contralateral ears. Scale bar: 100 μm.
